# Paralysis and Apoplexy

**Published:** 1890-11

**Authors:** O. S. Poston


### PARALYSIS AND APOPLEXY.

O. S. POSTON, IN "HEALTH AND HOME."

These diseases, when they proceed from lesions in the brain, are somewhat alike in the causes that produce them.

Two of the chief causes are meat eating and the use of intoxicating liquors, and often both combined. Every artery and vein in the brain, as elsewhere, is surrounded by a coat of muscular matter, and, so far as it envelopes the arteries, expand and contract with every pulsation of the heart. When one feeds on animal food or alcoholic drinks, all the muscles, including those surrounding the blood vessels, are more subject to be inflamed than if he fed on bread, vegetables and fruit diet. When those muscular coats become inflamed, they are increased in thickness and press upon the brain matter or break at some point, and a clot of blood exudes, and by its presence produces diseased condition, resulting in one or the other of the above maladies. Those who are predisposed to those diseases—who are fleshy and short-necked, or whose ancestors have died from those diseases—will be wise and prudent if they avoid the use of liquors that are intoxicating, and also the excessive use of animal food, including eggs. It will be advisable that they only partake of animal food once a day, and even then in moderate quantity.

Dr. Cheyne, who was a celebrated physician who resided in London last century, required all persons who had survived the first attack of paralysis or apoplexy to restrict themselves to a milk and bread diet, limiting them to not exceeding two quarts of skimmed milk and a small quantity of bread.

There is no doubt that a vegetarian diet is the best remedy against the recurrence of the maladies alluded to in this article.

In paralysis, alcoholic sponge baths at bed-time have been recommended as curative ; also, hand baths administered by a healthy operator by repeatedly dipping his hands in hot water and rubbing the patient freely, just before retiring. It imparts the magnetism of the operator more fully than any other method, and rouses up the circulation of the nervous forces of the patient more effectually than any medicine.

The hot hand bath, with proper restriction of diet, affords the best hope of recovery.

The first attack does not always prove fatal, but by prudence in diet it may not recur again.

